# *Pseudomonas aeruginosa* Modulates the Antiviral Response of Bronchial Epithelial Cells

**DOI:** 10.3389/fimmu.2020.00096

**Published:** 2020-02-04

**Authors:** Michael Sörensen, Julia Kantorek, Lauren Byrnes, Sébastien Boutin, Marcus A. Mall, Felix Lasitschka, Heike Zabeck, Dao Nguyen, Alexander H. Dalpke

**Affiliations:** ^1^Department of Infectious Diseases, Medical Microbiology and Hygiene, University Hospital Heidelberg, Heidelberg, Germany; ^2^Laboratory Enders and Partners, Stuttgart, Germany; ^3^Translational Lung Research Center Heidelberg (TLRC), German Center for Lung Research (DZL), University Hospital Heidelberg, Heidelberg, Germany; ^4^Department of Pediatric Pulmonology, Immunology and Intensive Care Medicine, Charité Universitätsmedizin Berlin, Berlin, Germany; ^5^Berlin Institute of Health (BIH), Berlin, Germany; ^6^Institute of Pathology, University Hospital Heidelberg, Heidelberg, Germany; ^7^TI Biobanking, German Centre for Infection Research (DZIF), Heidelberg, Germany; ^8^Thoraxklinik, University Hospital Heidelberg, Heidelberg, Germany; ^9^Meakins-Christie Laboratories, Research Institute of the McGill University Health Centre, Montreal, QC, Canada; ^10^Department of Medicine, McGill University, Montreal, QC, Canada; ^11^Institute of Medical Microbiology and Hygiene, Medical Faculty, Technische Universität Dresden, Dresden, Germany

**Keywords:** *Pseudomonas aeruginosa*, cystic fibrosis, virus, antiviral response, interferon, protease, LasR

## Abstract

Cystic fibrosis (CF) patients frequently acquire *Pseudomonas aeruginosa* infections that have been associated with a bad prognosis and an increased rate of pulmonary exacerbations. Respiratory viruses can cause exacerbations in chronic pulmonary diseases including COPD or asthma and have been suggested to contribute to exacerbations also in CF. In this study we investigated a possible link between *P. aeruginosa* infection and susceptibility to respiratory viruses. We show that *P. aeruginosa* is able to block the antiviral response of airway epithelial cells thereby promoting virus infection and spread. Mechanistically, *P. aeruginosa* secretes the protease AprA in a LasR dependent manner, which is able of directly degrading epithelial-derived IFNλ resulting in inhibition of IFN signaling. In addition, we correlate the virus infection status of CF patients with the ability of patients' *P. aeruginosa* isolates to degrade IFNλ. In line with this, the infection status of CF patients correlated significantly with the amount of respiratory viruses in sputum. Our data suggest that the interplay between *P. aeruginosa* and respiratory virus infections might partially explain the association of increased rates of pulmonary exacerbations and *P. aeruginosa* infections in CF patients.

## Introduction

Cystic fibrosis (CF) is an autosomal recessive hereditary disease caused by mutations in the Cystic Fibrosis Transmembrane Conductance Regulator (CFTR) gene. Mutations of CFTR lead to non- or mal-function of all exocrine glands and mucosal surfaces of the human body. Thus, the disease affects various organs including intestine, pancreas and lung (1). Life expectancy of CF patients is severely decreased and this is nowadays mainly dictated by the pulmonary phenotype: CF patients suffer from thickened respiratory mucus causing mucus plugging of the airways, chronic inflammation as well as increased incidence of pulmonary bacterial infections. Infections are of polymicrobial nature yet *H. influenzae, S. aureus*, and *P. aeruginosa* are clinically important pathogens in CF lung disease (2). Bacterial airway infection and inflammation associated with reduced mucociliary clearance mediate progressive lung damage and a decline in lung function over time, finally resulting in death due to respiratory failure.

Especially chronic airway infections with *P. aeruginosa* have been correlated with an accelerated loss of lung function (3, 4). *P. aeruginosa* infections typically start as intermittent infection with environmental strains that initially are sensitive to antibiotic eradication. However, over time *P. aeruginosa* undergoes adaptive mutations including gain of antibiotic resistance, loss of virulence factors, e.g., proteases or pyocyanin production, and increased alginate synthesis. This favors the establishment of chronic infection and resistance to antibiotic treatment that results in failure of eradication (5). Several secreted proteases of *P. aeruginosa* have been described modulating the inflammatory response of the host. As such, LasB, a protease under the control of the quorum sensing receptor LasR, has been demonstrated to degrade IL-6 and IL-8. This helps *P. aeruginosa* to establish an infection since it blocks the recruitment of leukocytes (6). Also other LasR regulated proteases (7, 8), like LasA or AprA, have been demonstrated to degrade cytokines and might act in the same way as LasB (9). Interestingly, as soon as *P. aeruginosa* infection has been established, LasR often acquires loss of function mutations during the transition of intermittent to chronic infections and thereby further boosts pulmonary inflammation (6).

However, the decline in lung function that is associated with development of chronic infection with *P. aeruginosa* is not constant or linear. Instead, periods of relatively stable lung function are interrupted by episodes with an acute drop in lung function, from which full recovery might not be achieved by antibiotic treatment (10). Causes and pathological mechanisms involved in these pulmonary exacerbations are often unclear and bacterial and viral infections have been attributed to it (11). Virus-induced pulmonary exacerbations are well-known in other lung diseases like COPD or asthma (12). Yet, the importance of viral induced pulmonary exacerbations in CF patients is still unclear (13, 14). However, it has been shown that the lung microbiome composition itself is quite resilient and does not change to great extent in most cases of exacerbation (15, 16) and therefore the involvement of non-bacterial organisms, including viruses, is likely. The antiviral response is triggered by intracellular recognition of viruses via nucleic acid pattern receptors including TLR3 and RIG-I. Activation of these receptors induces an initial type I/III IFN synthesis which subsequently boosts its own production in a positive feedback loop (17). It has been shown that respiratory epithelial cells produce mainly type III IFN and the importance of these proteins in the airways is well-documented (18, 19). Moreover, manipulation of type III IFN has been linked to increased susceptibility of asthmatic patients toward human rhinoviruses (hRV) and a contribution to pulmonary exacerbation has been suggested (20, 21). Since *P. aeruginosa* and respiratory viruses have been linked to pulmonary exacerbations and in addition, respiratory viruses have been associated with the transition from transient to chronic airway infections with *P. aeruginosa* (22, 23) a link between both microorganisms is likely. Therefore, we investigated in this study if *P. aeruginosa* is able to modulate the antiviral response of bronchial epithelial cells and how such interplay might happen at the mechanistical level. In addition we analyzed sputa of CF patients for the presence of respiratory viruses and determined the levels of virus RNA in order to link *P. aeruginosa* to virus infection thus identifying clinical importance of the experimental findings.

## Results

### *P. aeruginosa* Inhibits the Antiviral Response of Airway Epithelial Cells

**Figure 1:**
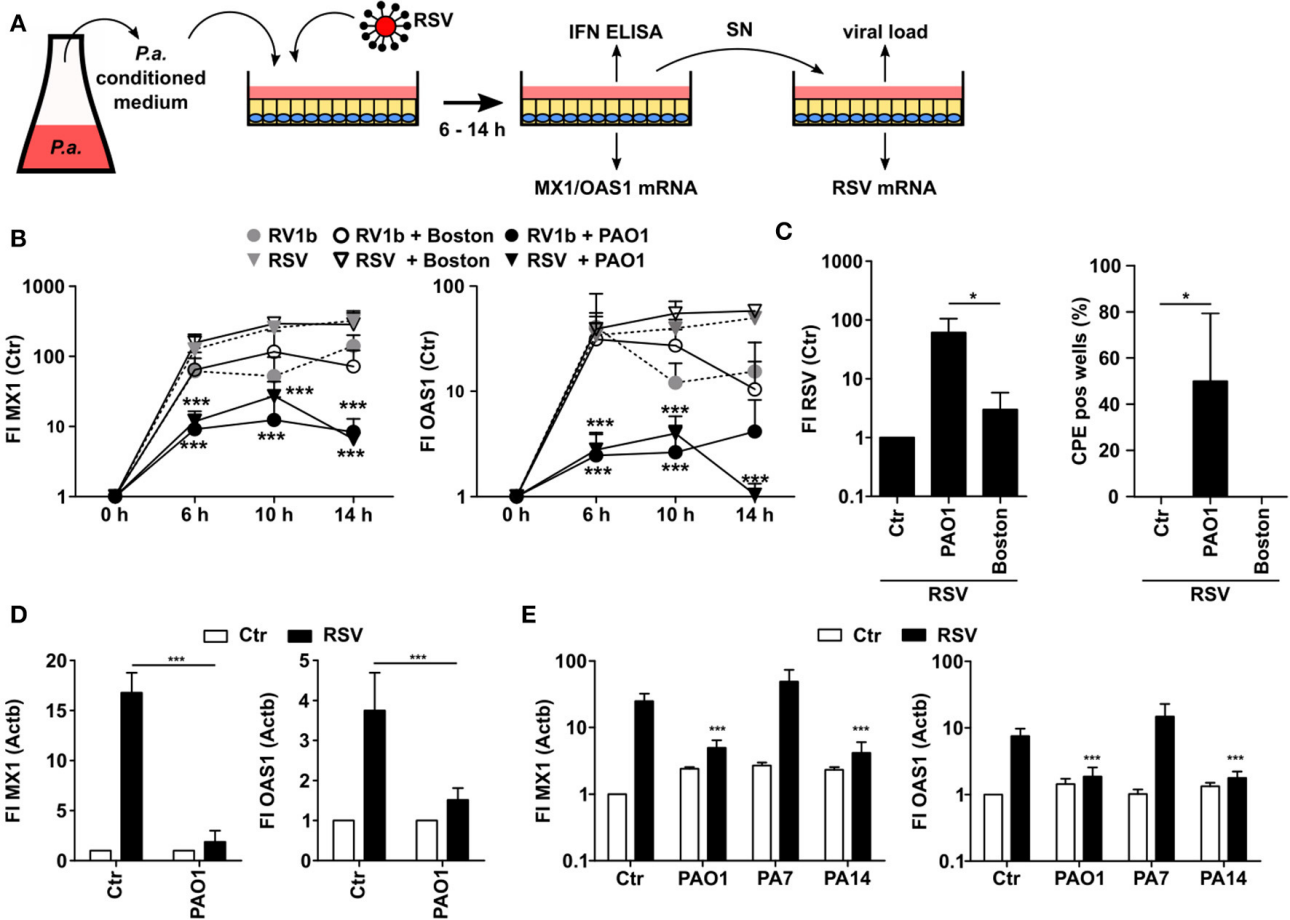
*P. aeruginosa* is able to suppress antiviral response of airway epithelial cells. **(A)** Cell culture based experimental workflow. **(B)** BEAS2B cells were pretreated with conditioned medium of *P. aeruginosa* (PAO1 and Boston) or control medium and subsequently infected with RSV or RV1b. Induction of MX1 and OAS1 mRNA were analyzed by qRT-PCR after 6, 10, and 14 h. **(C)** BEAS2B cells were treated as in **(A)** and cell culture supernatant of RV1b (human RV) or RSV infected cells (14 h) was transferred to new BEAS2B cells. Levels of RV1b (hRV) or RSV mRNA were analyzed by qRT-PCR and the number of wells displaying RV1b or RSV specific cytopathogenic effects (CPE) was determined. **(D)** Primary HBE cells were pretreated with conditioned medium of *P. aeruginosa* (PAO1) or control medium and subsequently infected with RSV. Induction of MX1 and OAS1 mRNA were analyzed by qRT-PCR after 14 h. **(E)** BEAS2B cells were pretreated with conditioned medium of *P. aeruginosa* or control medium and subsequently infected with RSV. Induction of MX1 and OAS1 mRNA were analyzed by qRT-PCR after 14 h. All experiments *n* = 3–4, ANOVA and Bonferroni post-test was used for statistical analysis. Significant differences were considered at ^*^p < 0.05, ^**^p < 0.01, and ^***^p < 0.001 as compared to the control condition. n.s., not significant.

In order to analyze whether *P. aeruginosa* is able to modulate the antiviral response of bronchial epithelial cells, we prepared control medium or conditioned medium (CM) from two different strains of *P. aeruginosa*, PAO1 (commonly used in research) and Boston (quality control strain), respectively. CM contains soluble factors secreted by *P. aeruginosa* during growth. We focused on soluble factors since *P. aeruginosa* is mostly located intraluminally in CF lungs and direct cell-cell contacts are less common (24). Subsequently, we used CM or control medium to treat airway BEAS2B cells and thereafter infected the cells with hRV (strain RV1b) or RSV (Figure 1A). Subsequently, antiviral responses were analyzed after various incubation times. Induction of the antiviral genes MX1 and OAS1 upon virus infection did not show any significant differences between BEAS2B cells pretreated with CM of *P. aeruginosa* Boston strain (CM-Boston) or control medium (Figure 1B). However, cells treated with CM of *P. aeruginosa* strain PAO1 (CM-PAO1) showed a significant decrease in the induction of both genes after infection with hRV and RSV, which was most pronounced after 14 h of infection compared to control medium [fold induction (FI) of MX1: 285 vs. 6, *p* < 0.001; FI OAS1: 49 vs. 1, *p* < 0.001; Figure 1A]. Since effects were stronger after RSV infection all subsequent experiments were done with RSV. Of note, conditioning with *P. aeruginosa* medium alone did not affect MX1 or OAS1 expression (not shown).

As *P. aeruginosa* might act cytotoxic to eukaryotic cells we chose to use only conditioned medium instead of direct bacterial infection and we also controlled viability by annexin V and propidium iodide staining. Even though we observed some cytotoxic effects at 14 h we did not detect differences in viability of epithelial cells conditioned with medium from either of the two *P. aeruginosa* strains up to 10 h (viability was 96 ± 1.7% for Boston vs. 96 ± 2.7% for PAO1 after hRV infection and 95 ± 2% and 96 ± 3% after RSV infection, respectively). Therefore, cytotoxic effects can be excluded as an explanation why only PAO1 inhibited the antiviral response. Of note, we did not observe any differences by any CM treatment with respect to the viral load of the primary infected cells indicating that the first infection of the cells was not affected by soluble *P. aeruginosa* factors (Figure S1). Yet, we speculated that the inhibition of the antiviral response by CM-PAO1 (as indicated by reduced ISG induction) may facilitate spread of the virus infection. To analyze the functional relevance of the decreased antiviral response, we therefore challenged another batch of BEAS2B cells with the supernatant of CM-pretreated and RSV infected cells (Figure 1A). In line with a decrease of the antiviral response in CM-PAO1 treated BEAS2B cells, we observed increases in RSV-RNA and RSV-specific cytopathic effects in cells challenged with the supernatant of CM-PAO1 treated cells as compared to control or CM-Boston treated cells (Figure 1C). These results indicate that the repression of the antiviral response by PAO1 leads to an increase in virus spreading and is therefore of functional relevance. To confirm the effects in primary cells we repeated the main experiment in primary human bronchial epithelial cells (hBrEpC). In line with our observations in BEAS2B cells, PAO1 was also able to repress RSV induced antiviral responses in hBrEpC (Figure 1D). It is known that *P. aeruginosa* can be classified into three different phylogenetic groups (25), represented by PA14, PAO1, and PA7 for group 1, 2, and 3, respectively. Like PAO1 PA14 but not PA7 was also able to repress the antiviral response toward RSV (Figure 1E) indicating that the inhibition of an antiviral response is not restricted to a specific phylogenetic group.

### *P. aeruginosa* Blocks IFNλ Activity

**Figure 2:**
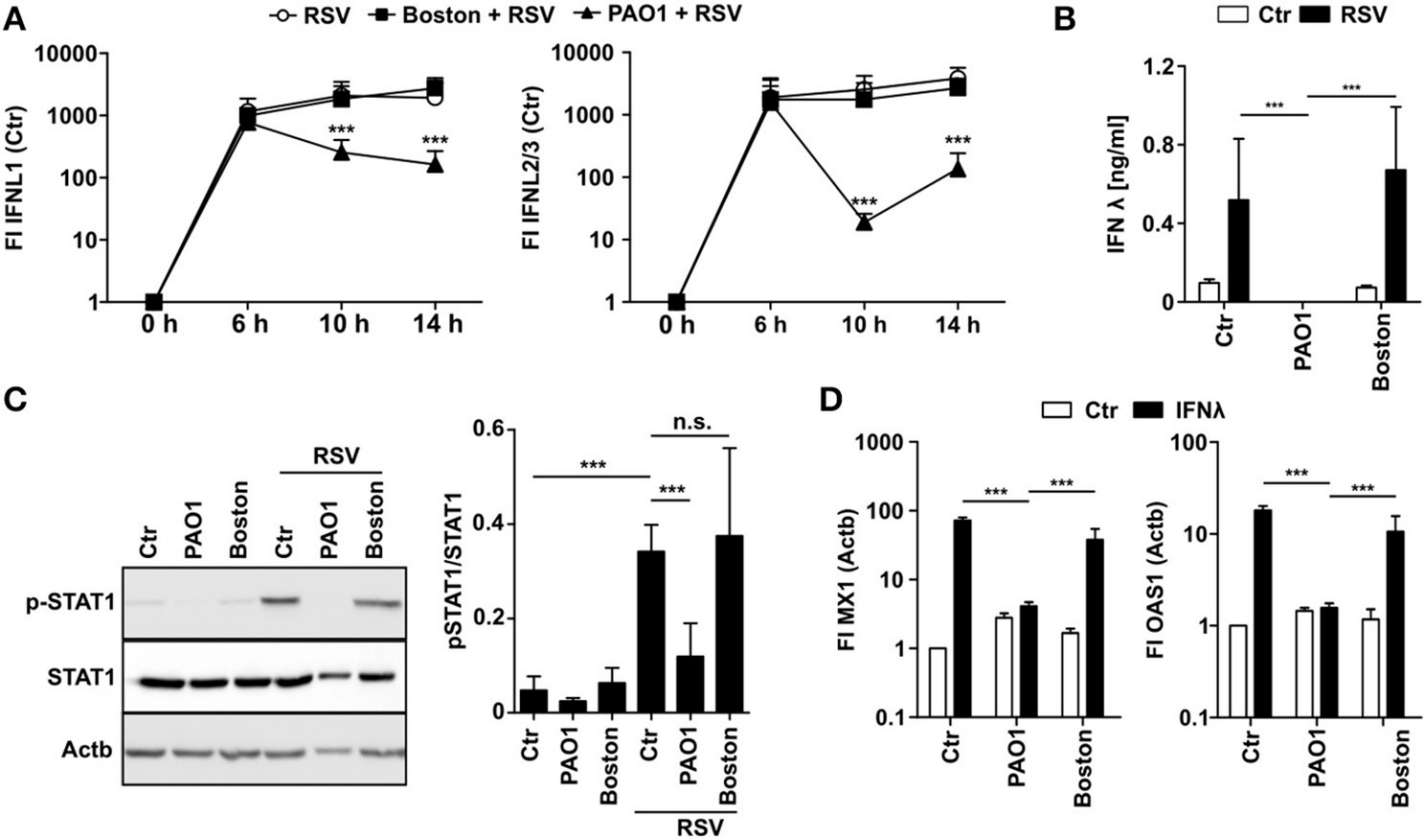
*P. aeruginosa* is able to block IFN induction and signaling. **(A)** BEAS2B cells were pretreated with conditioned medium of *P. aeruginosa* (PAO1 and Boston) or control medium and subsequently infected with RSV. Induction of IFNL1 and IFNL2/3 mRNA were analyzed by qRT-PCR after 6, 10, and 14 h. **(B)** BEAS2B cells were treated as in **(A)** and IFNλ levels of cell culture supernatant (14 h) were analyzed by ELISA. **(C)** BEAS2B cells were treated as in **(A)** and p-STAT1 levels of cells (6 h) were analyzed in cell lysates by western blot. **(D)** BEAS2B cells were pretreated as in **(A)**. Cells were then stimulated with recombinant IFNλ (5,000 pg/ml) and induction of MX1 and OAS1 were analyzed after 14 h by qRT-PCR. All experiments *n* = 3–4, ANOVA and Bonferroni post-test was used for statistical analysis. Significant differences were considered at ^*^p < 0.05, ^**^p < 0.01, and ^***^p < 0.001 as compared to the control condition. n.s., not significant.

To elucidate how PAO1 inhibits induction of antiviral gene expression, we first analyzed IFN production in PAO1-CM treated cells after infection with RSV. IFNλ is the main IFN produced by airway epithelial cells upon viral infections, whereas RSV did not induce IFNα mRNA and only moderate levels of IFNβ mRNA (Figure S2). Interestingly, early IFNλ mRNA levels at 6 h p.i. were similar in control or CM-treated cells suggesting that initial virus recognition was not affected. However, at 10 and 14 h p.i. IFNλ mRNA levels decreased significantly in PAO1-CM treated cells (Figure 2A). Importantly, virtually no IFNλ protein was detectable in the cell culture supernatant of PAO1-CM treated cells at 14 h (Figure 2B) indicating that despite an early mRNA induction, positive amplification, and final production of IFNλ protein was blocked. For virus infection we were not able to detect functional IFNβ protein (data not shown). These findings may be explained by PAO1 modulating IFNλ protein production, secretion, stability, or signaling, thereby blocking the secondary positive feedback loop important for sustained and efficient IFN signaling. In line, STAT1 phosphorylation as an endogenous indicator of IFN signaling was missing in the presence of PAO1-CM but not with Boston-CM (Figure 2C). Next, we tested if recombinant exogenous IFNλ could overcome PAO1-CM effects as should be the case if production and secretion would be hampered: Surprisingly, addition of exogenous IFNλ resulted in MX1 and OAS1 induction in control cells and Boston-CM treated cells, yet in PAO1-conditioned BEASB cells no induction could be observed (Figure 2D). The results indicated that PAO1 could either block the recognition of IFNλ by its receptor or destabilize IFNλ protein.

### *P. aeruginosa* PAO1 Degrades IFNλ by Secretion of Proteases

**Figure 3:**
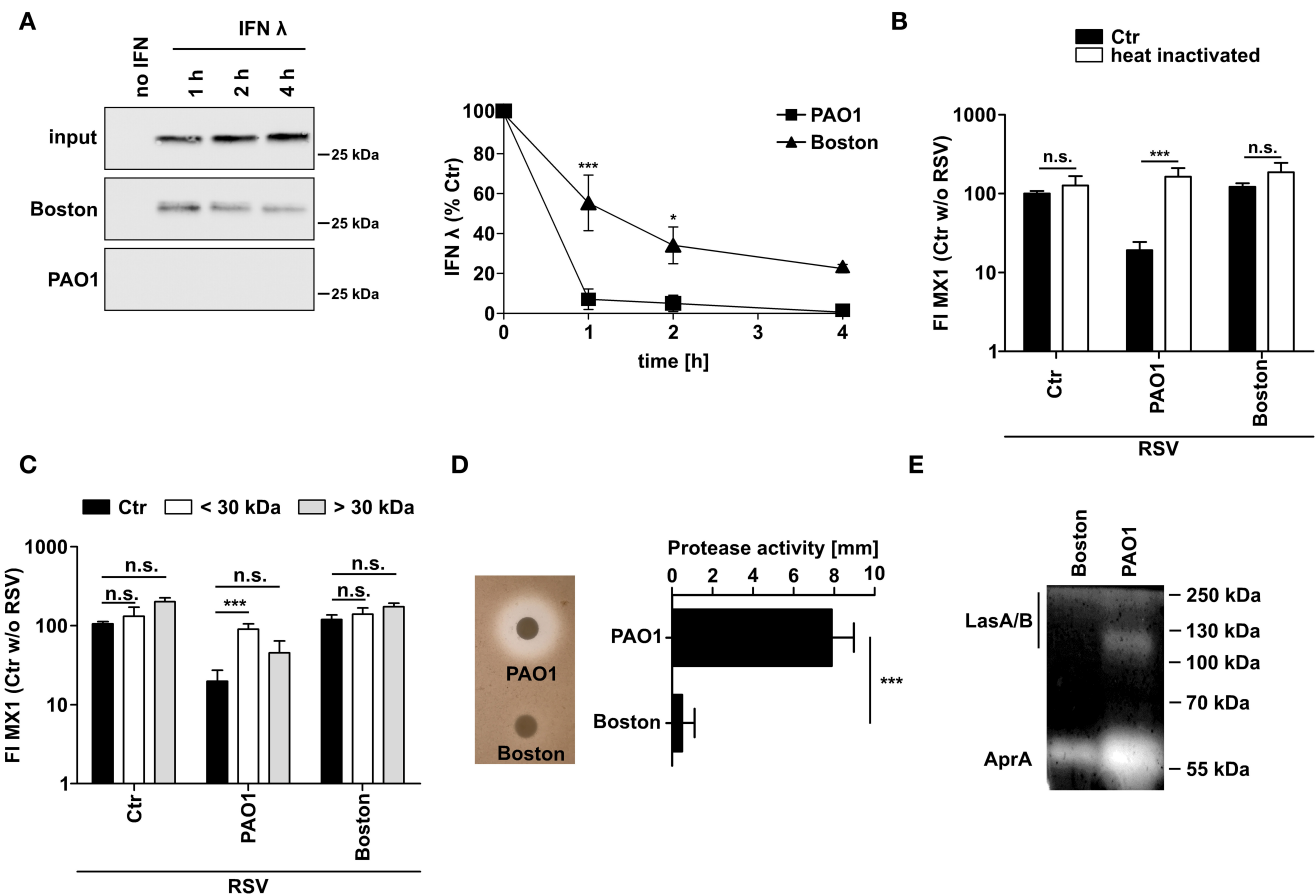
*P. aeruginosa* secretes a protease degrading directly IFNλ. **(A)** Recombinant IFNλ was incubated in conditioned medium of *P. aeruginosa* (PAO1 and Boston) or control medium at 37°C for 1, 2, or 4 h. Subsequently, levels of IFNλ were analyzed by western blot. **(B)** Conditioned media was heated for 10 min at 95°C. Subsequently BEAS2B cells were pretreated with heat treated or non-treated conditioned medium of *P. aeruginosa* (PAO1 and Boston) or with control medium and subsequently infected with RSV. Induction of MX1 mRNA was analyzed by qRT-PCR after 14 h post-infection. **(C)** Conditioned media was filtered using a cutoff of 30 kDa. Subsequently BEAS2B cells were pretreated with the fraction larger than 30 kDa, smaller than 30 kDa, non-filtered conditioned medium of *P. aeruginosa* (PAO1 and Boston) or control medium and subsequently infected with RSV. Induction of MX1 mRNA was analyzed by qRT-PCR after 14 h post-infection. **(D)** Protease activity in conditioned medium of *P. aeruginosa* (PAO1 and Boston) was assessed using skim-milk agar. **(E)** Elastase activity in conditioned medium of *P. aeruginosa* (PAO1 and Boston) was assessed by zymography. All experiments *n* = 3–4, ANOVA and Bonferroni post-test was used for statistical analysis. Significant differences were considered at ^*^p < 0.05, ^**^p < 0.01, and ^***^p < 0.001 as compared to the control condition. n.s., not significant.

Since *P. aeruginosa* is known to secret various proteases, we next tested whether CM of *P. aeruginosa* is able to degrade recombinant IFNλ directly (Figure 3A). CM of both strains, PAO1 and Boston, degraded recombinant IFNλ but PAO1 was much more efficient with complete destruction of added IFNλ even after 1h of incubation. To elucidate the mechanism of action, we heat treated (10 min, 95°C) or filtered (molecular weight cut-off 30 kDa) the conditioned medium from both *P. aeruginosa* strains (Figures 3B,C). Both treatments were able to restore MX1 and OAS1 induction by RSV in PAO1-conditioned cells indicating that the inhibitory factors in PAO1-CM might be proteins of <30 kDA. We next analyzed the general secretion of proteases by both strains using casein degradation (Figure 3D) or zymography (Figure 3E). Although both strains showed protease secretion, protease activity was much higher in PAO1 (Figure 3D). Zymography also showed that PAO1 had higher AprA and LasA/B activity (as determined by size) whereas the Boston strain only expressed low amounts of AprA (Figure 3E).

### *P. aeruginosa* Isolates From CF Patients With Intermittent, but Not With Chronic Infection Show Inhibition of Antiviral Responses

**Figure 4:**
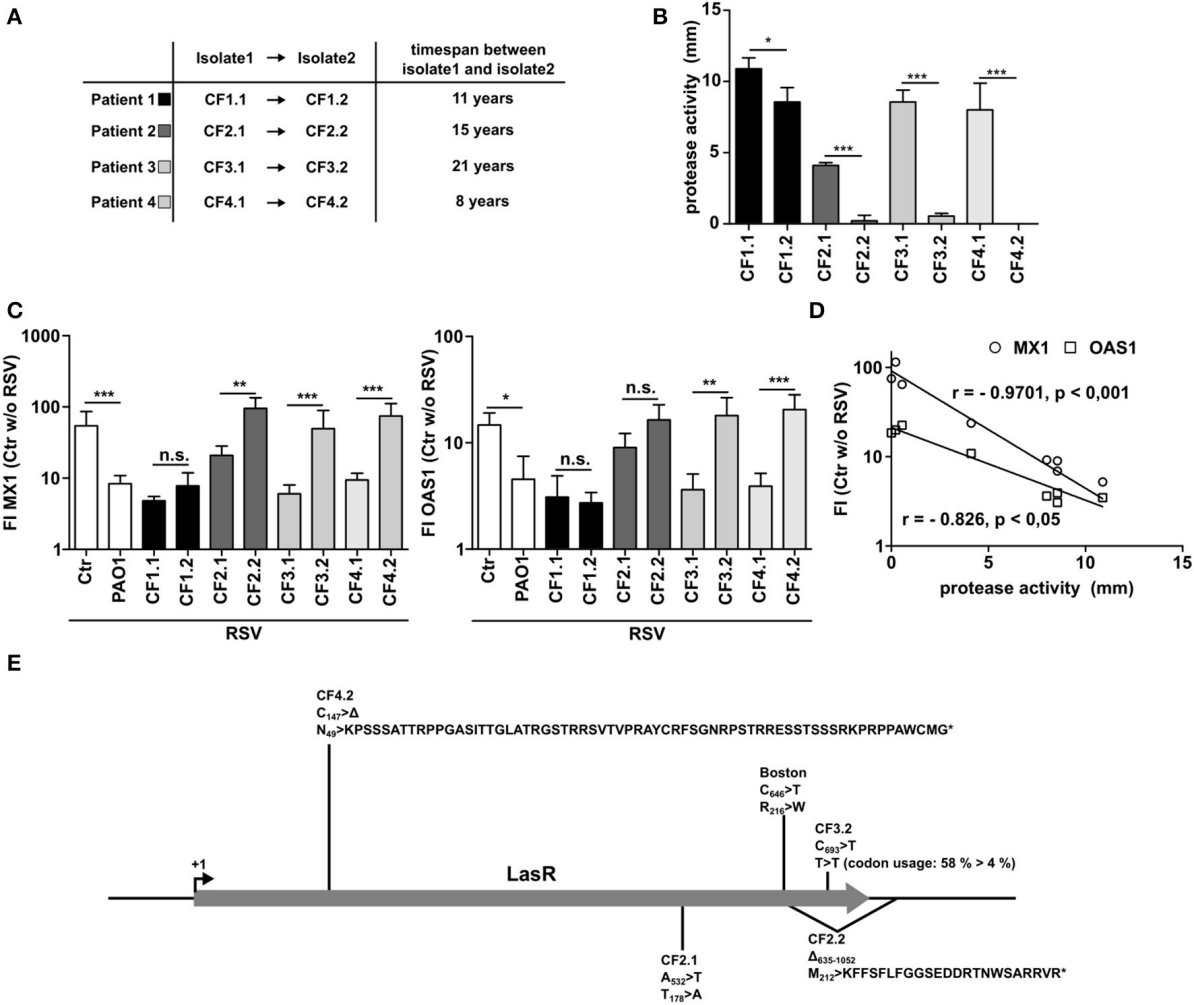
Late *P. aeruginosa* CF isolates display low protease activity and decreased ability to inhibit antiviral response. **(A)** Characteristics of longitudinal isolates of *P. aeruginosa* from CF patients. **(B)** Protease activity of *P. aeruginosa* CF isolates was assessed using skim milk agar. **(C)** BEAS2B cells were pretreated with conditioned medium of *P. aeruginosa* from **(A)** or control medium and subsequently infected with RSV. Induction of MX1 and OAS1 mRNA were analyzed by qRT-PCR after 14 h. **(D)** Spearman correlation of protease activity and induction of MX1 or OAS1 mRNA measured in **(B,C)**. **(E)** LasR genetic mutations and resulting changes in protein translation found in CF isolates described in **(A)**. All experiments *n* = 4, ANOVA and Bonferroni post-test was used for statistical analysis. Significant differences were considered at ^*^p < 0.05, ^**^p < 0.01, and ^***^p < 0.001 as compared to the control condition. n.s., not significant.

It is well-known that *P. aeruginosa* adapts to the lung microenvironment in CF patients by downregulating several virulence factors over time including secreted proteinases when establishing a chronic infection. Therefore, we made use of four longitudinal *P. aeruginosa* pairings from four different CF patients (CF1 to CF4). All pairings are clonally related as was shown by RFLP before (6) and were isolated from the same patients with at least 8 years time span (Figure 4A). Whereas, the early isolates of each patient showed considerable protease activity, the late-stage isolates had significantly lower protease activity (Figure 4B). Analyzing the effect of the early isolates on the antiviral response to RSV, all isolates were able to suppress the induction of MX1 or OAS1. In line with their reduced protease activity, 3 out of 4 of later isolates displayed a lower or completely missing capacity to suppress the antiviral response (Figure 4C). An exception was isolate CF1.2, but as shown in Figure 4B this isolate also showed only a minor reduction in protease activity. Of note, expression of MX1 or OAS1 correlated significantly with the protease activity measured (Figure 4D). Many of the secreted proteases are under the regulation of the transcription factor LasR. Since LasR is known to become mutated in progressed stages of *P. aeruginosa* infection in CF patients (26), we sequenced LasR of all isolates used (CF1-4). We identified mutations in the late-stage isolates and in *P. aeruginosa* Boston (Figure 4E). Those mutated strains were the strains that had no inhibitory effect on MX1 and OAS induction (Figure 4C). Of note, no mutation of LasR was identified in CF1.2, the only strain that was still able to inhibit the antiviral response. Additionally, a mutation in LasR was detected in the early strain CF2.1 that was the one with low protease activity even at early time of infection (Figure 4B). The mutations were either non-synonymous mutations (Boston, CF2.1), deletions resulting in a frameshift and nonsense peptide sequence (CF2.2, CF4.2) or a synonymous mutation resulting in a seldom-used codon/t-RNA combination for *P. aeruginosa* (CF3.2) (Figure 4E). In line with this, PA7, a group 3 *P. aeruginosa* strain (also not able to suppress antiviral response, Figure 1E), has only 93% identical nucleotides compared to the reference strain PAO1 which results in three amino acid exchanges (K_70_ >R; S_142_ >N; N_182_ >S).

### Inhibition of the Antiviral Response by *P. aeruginosa* Depends on LasR and AprA

**Figure 5:**
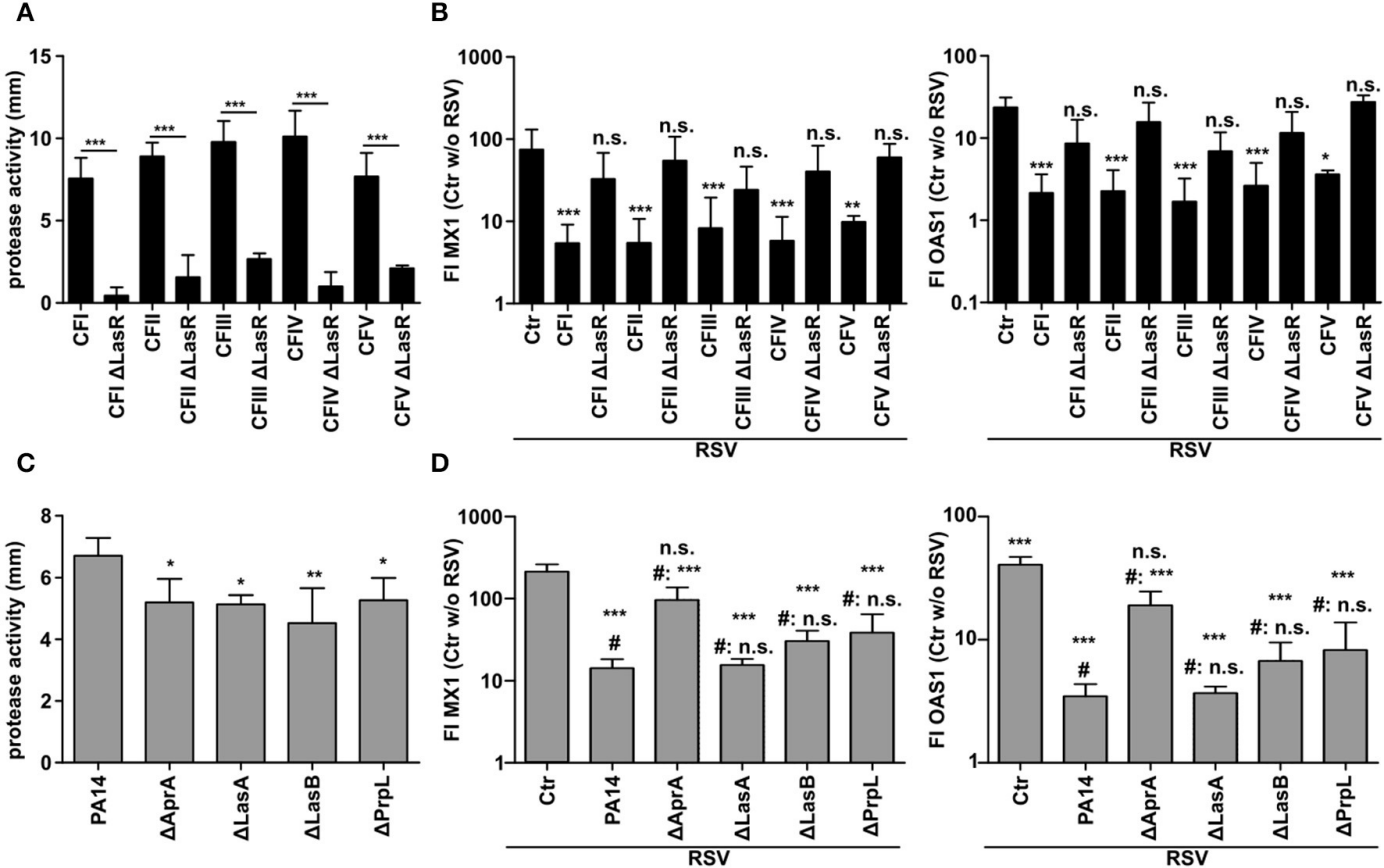
Inhibition of antiviral response by *P. aeruginosa* depends on quorum sensing and the secreted protease AprA. **(A)** Protease activity of *P. aeruginosa* CF isolates and corresponding LasR deficient strains was assessed using a skim milk agar assay. **(B)** BEAS2B cells were pretreated with conditioned medium of *P. aeruginosa* from **(A)** or control medium and subsequently infected with RSV. Induction of MX1 and OAS1 mRNA were analyzed by qRT-PCR after 14 h. **(C)** Protease activity of *P. aeruginosa* strain PA14 and corresponding protease deficient strains was assessed using skim milk agar. **(D)** BEAS2B cells were pretreated with conditioned medium of *P. aeruginosa* from **(C)** or control medium and subsequently infected with RSV. Induction of MX1 and OAS1 mRNA were analyzed by qRT-PCR after 14 h. All experiments *n* = 4–5, ANOVA and Bonferroni post-test was used for statistical analysis. **(D)** Statistical analysis was done in comparison to the control (ctr) or to treatment with PA14 (indicated by prefix #). Significant differences were considered at ^*^p < 0.05, ^**^p < 0.01, and ^***^p < 0.001 as compared to the control condition. n.s., not significant.

Since the previous data implied an involvement of the quorum sensing protein LasR, we again used further clinical CF isolates (CFI-V) and their counterparts with a targeted LasR mutation. In line with the reported importance of LasR in the expression of proteases (6) all LasR deficient CF isolates displayed significantly lower protease activity in CM (Figure 5A). In parallel, all CF isolates with functional LasR were able to suppress the induction of MX1 and OAS1 after RSV infection whereas their LasR deficient counterparts were not (Figure 5B). The most important proteases regulated by LasR are AprA, LasA, LasB, and PrpL. In order to investigate which LasR dependent protease is involved in the modulation of the antiviral response, we used strain PA14 and transposon mutants of PA14 with specific protease deficiency. We observed that even though all protease deficient mutants showed a decrease in total protease activity in CM (Figure 5C), only AprA-deficient PA14 was not able anymore to suppress the induction of antiviral genes MX1 and OAS1 (Figure 5D). These observations indicate that the LasR-regulated protease AprA degrades IFNλ, thereby blocking the induction of the antiviral response upon RSV infection.

### CF Patients With Intermittent *P. aeruginosa* Infection Are at Higher Risk to Be Infected With Human Rhinovirus

**Figure 6:**
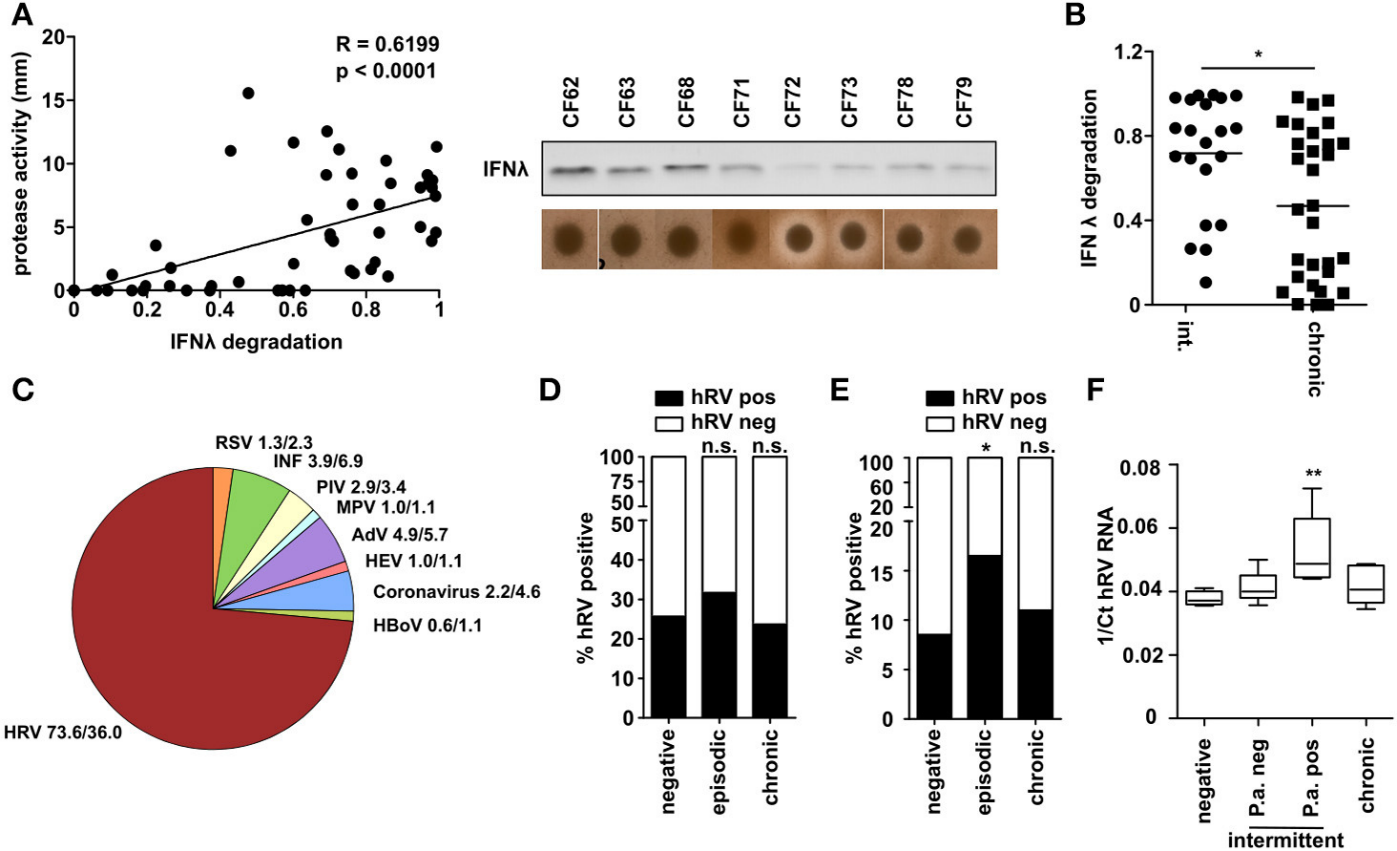
CF patients with intermittent *P. aeruginosa* infection are at higher risk to be infected with hRV. **(A)** Capacity of CF *P. aeruginosa* isolates to degrade IFNλ was analyzed by western blot and protease activity was measured by skim milk agar. Correlation was assessed using Spearman analysis (*n* = 51). **(B)** *P. aeruginosa* isolates from **(A)** were classified according to infection status and plotted against relative IFNλ degradation after 1 h. Statistical analysis was done using non-parametric Mann-Whitney test. **(C)** Presence of respiratory viruses in sputum samples of CF patients were assessed using a multiplex diagnostic virus panel assay. Relative occurrence of respiratory virus positive samples and total samples are indicated (*n* = 340). **(D)** The prevalence of hRV in CF samples (nose, throat and sputum) was plotted according to the infection status (*n* = 340). Statistics was done by using Fisher's Exact Test. **(E)** The prevalence of hRV in CF sputum samples was plotted according to the infection status (*n* = 499). Statistics was done by using Fisher's Exact Test. **(F)** Presence of hRV in sputum from CF patients was assessed by qPCR. The Ct values of hRV was plotted according to the infection status (*n* = 24). Significant differences were considered at ^*^p < 0.05, ^**^p < 0.01, and ^***^p < 0.001 as compared to the control condition. n.s., not significant.

To examine the biological significance of the above findings, we next analyzed a collection of *P. aeruginosa* isolates obtained from CF patients at various stages of *P. aeruginosa* infection. We measured total protease activity of *P. aeruginosa* isolates and their ability to degrade recombinant IFNλ. We observed a strong correlation (*R* = 0.62) and significant interdependency between protease activity and IFNλ degradation (Figure 6A). Moreover, when grouping the *P. aeruginosa* isolates according to their stage of infection (intermittent/early or chronic infection/late) we observed that especially *P. aeruginosa* of intermittent infected patients were able to degrade IFNλ (Figure 6B). This could indicate that especially intermittent infected CF patients are prone to acquire respiratory virus infections. Therefore, we checked which viruses are present in respiratory material (nose or throat swabs, sputum) of CF patients (Figure 6C). Using a multiplex diagnostic virus panel, we screened 818 samples of 526 visits. We could detect viruses in 162 samples (30.85%). Most of the viruses found in these patients were human rhinoviruses (hRV) (66%) whereas RSV was only found to a lower extent and at similar frequency to Influenza, Adenovirus or Parainfluenza Virus (3.7–7.4%). Since hRV was the most prevalent virus detected and *P. aeruginosa* was also able to suppress hRV induced antiviral response (Figure 1B), we decided to further analyze the presence of hRV in the sputum of these patients, since *P. aeruginosa* can be reliably detected in this material. In accordance with the literature we did not detect any difference between the three groups if all samples (nose, throat, and sputum) from one patient were considered (Figure 6D). However, restriction of the analysis to lower airways' sputum samples from CF patients (*n* = 499) revealed that the prevalence of hRV detection was significantly higher (*p* = 0.037) in intermittent *P. aeruginosa* infected CF patients (16.5%) than in chronic patients (8.5%) or in patients not infected with *P. aeruginosa* (11.0%) (Figure 6E). In line with this, the detectable virus load indicated by the hRV Ct value by RT-PCR was highest in samples of intermittent infected CF patients, which were culture-positive for *P. aeruginosa* at the time of sampling (Figure 6F). These observations in clinical samples indicate that *P. aeruginosa* may also be able to modulate the antiviral response *in vivo* in the clinical setting of CF.

## Discussion

*Pseudomonas aeruginosa* is one of the most important pathogens in the context of CF (2). Infections with *P. aeruginosa* can be distinguished into two different groups: (i). intermittent infections in which the infection with *P. aeruginosa* can be cleared by antipseudomonal treatment, (ii). chronic infections in which *P. aeruginosa* can be isolated from most of the respiratory samples indicating that the respiratory tract is chronically colonized by this bacterium (26). Infections with *P. aeruginosa* are correlated with an accelerated decline in lung function and with an increased incidence of respiratory exacerbations (27, 28). Viral infections have been linked to exacerbations in other chronic pulmonary diseases and respiratory virus infections in CF patients seem to be associated with higher viral burden and higher morbidity (29). Investigation of the link between *P. aeruginosa* and respiratory viruses could therefore identify potential new therapeutic and diagnostic strategies.

We infected human bronchial epithelial cells (BEAS2B and human primary bronchial epithelial cells) with RSV or hRV in the presence of *P. aeruginosa* conditioned medium. We used conditioned medium to analyze soluble, secreted factors from *P. aeruginosa* that might interfere with sensitivity to virus infection. Preliminary experiments (data not shown) indicate that also infection with live *P. aeruginosa* results in inhibition of type III IFN activity and thus recapitulates the effects with conditioned medium. We observed that PAO1 was able to suppress the antiviral response of bronchial epithelial cells toward respiratory viruses (RSV and hRV) which subsequently lead to higher virus titers in secondary infected cells. Due to the stronger effects observed with RSV we subsequently focused on RSV modulation, but findings were similar for hRV (Figures S3–S5). Interestingly, *P. aeruginosa* strain Boston, a control strain frequently used in *Pseudomonas* research was not able to modulate the antiviral response. Due to genetic heterogeneity, *P. aeruginosa* can be divided into three phylogroups (25). Using representative strains of each group (PAO1—group 1; PA14—group 2; PA7—group 3) we observed that only the minor group 3 strain PA7 was not able to modulate the antiviral response, whereas PA14 could, a clone for which global spread has been shown (25, 30). These findings indicate that most of the *P. aeruginosa* strains found in the environment and causing infections in CF patients are able to modulate the antiviral response since most of *P. aeruginosa* isolates are of group 1 or group 2. Two earlier studies could also demonstrate that *P. aeruginosa* is able to modulate the antiviral response of epithelial cells (31, 32). In addition, the first study could demonstrate a difference in virus-induced IFN expression between healthy and CF-derived cells. However, we and the second study did not observe differences in IFN induction. In line with our results, a third study also failed to detect a difference of virus-induced IFN production between healthy and CF-derived epithelial cells (33). Therefore, our observations might also be relevant in diseases where chronic *P. aeruginosa* infections occur and a role of respiratory viruses in pulmonary exacerbations has been established, e.g., COPD (34). We have not analyzed whether such modulatory activities are also realized in other lung pathogens, but so far the here identified mechanisms are only reported for *P. aeruginosa*. It has been reported that *P. aeruginosa* PAK1 infection in a mouse model can induce IFNλ which promotes an inflammatory response and has a negative impact on *P. aeruginosa* defense *in vivo* (35). Of note, PAK1 compared to PAO1 has a point mutation in LasR which might affect protease activity (36). Moreover, the PAK strain induced IFNβ in airway epithelial cells, but CF epithelial cells showed a reduced response compared to healthy cells (37). However, so far it had not been reported that CF epithelial cells display increased sensitivity toward viral infection (38) which was the main focus of our study.

The antiviral response can be subdivided into two stages. First, viruses get recognized by nucleic acid receptors that drive the expression of type I and III interferons. Subsequently, secreted IFN I/III activates the canonical transcription factor STAT1/STAT2/IRF9, which in a positive feedback loop again drives further IFN I/III expression plus additional antiviral genes like OAS1/2 or MX1 (39). Interestingly, the initial induction of IFNλ mRNA after virus infection was not altered by PAO1-CM, but nevertheless IFNλ protein and signaling was significantly reduced compared to control or Boston-CM treated cells. Of note and in line with the literature, type I IFN was not induced by RSV in bronchial epithelial cells (Figure S2). Further analysis revealed that *P. aeruginosa* secretes proteases degrading type III IFN and thereby inhibiting the antiviral response. Of note, we observed that also exogenous type I IFN was degraded (Figure S6) indicating that in a physiological setting type I IFN as produced by immune cells would also get inactivated. Moreover, protease activity of various *P. aeruginosa* CF isolates correlated significantly with the ability to degrade recombinant IFNλ. Most of the secreted proteases are under the control of the quorum sensing regulator LasR and we could demonstrate that the ability of PAO1, Boston or the longitudinal CF isolates to suppress the antiviral response was associated with functional LasR. It is well-known that LasR is subject to mutations in the course of *P. aeruginosa* infections in CF patients e.g., it was reported that in a CF cohort 22% of the *P. aeruginosa* strains have an altered LasR sequence (6, 25, 40, 41). The involvement of LasR is further supported by the fact that LasR deleted CF *P. aeruginosa* isolates were not able to modulate the antiviral response whereas their parental counterpart did. LasR dependent proteases contributing substantially to the virulence of *P. aeruginosa* are AprA, LasA, LasB, and PrpL (42, 43). A limitation of the study is that LasR complemented mutants could not be used. However, five independent targeted mutants behave exactly the same way and the loss of the ability to degrade IFNλ correlated with a mutated LasR. Using *P. aeruginosa* PA14 deleted of either of these proteases showed that AprA is mostly responsible for the modulation of the antiviral response. In line with this, comparison of the protein sequence of AprA of PAO1, PA14, and PA7 revealed that PA7/group 3 AprA did not cluster within PA14 or PAO1 (Figure S7). AprA, also known as serralysin or alkaline metalloprotease, is a metalloprotease regulated directly by LasR and has previously been reported to degrade complement, alpha1-proteinase inhibitor, interleukins and interferon gamma (6, 44, 45). It is secreted as an inactive zymogen, which becomes active by the cleavage of a 9-amino acid propeptide either by other proteases (LasA/B) or in an autocatalytic manner. To our knowledge this is the first study showing that AprA is also able to degrade IFNλ thereby modulating the antiviral response of epithelial cells. It is well-known that CF patients produce antibodies against several *Pseudomonas* antigens including AprA. Moreover, it has been shown that these antibodies are able to block AprA activity (46–49). These antibodies would therefore be able to counteract AprA dependent type III IFN degradation. However, these antibodies need to be present at high titers at the site of infection in the conducting airways. Since high titers are regularly detected only in chronically infected patients neutralizing antibodies are only present when AprA expression is decreased. In addition, anti AprA antibodies are IgG subtypes which get passively secreted in the alveolar space and subsequently transported by the mucocilliary escalator to the airways (50, 51). Considering decreased mucocilliary clearance in CF patients sufficient titers might not be reached in this condition. In line with our results, Bomberger et al. were able to show that CFTR inhibitory factor (CIF), secreted by *P. aeruginosa*, is able to block presentation of viral antigens on MHC class I of bronchial epithelial cells and recognition by CD8^+^ cells adding another layer of complexity on how *P. aeruginosa* is able to modulate the antiviral defense (52).

Chronic infection with *P. aeruginosa* in CF is subject to a complex adaptation to the CF lung leading to increased biofilm production and a decrease in the expression of various virulence factors, including secreted proteases (26, 53). In line with this, total protease activity of *P. aeruginosa* isolates derived from CF patients correlated with their potential to degrade IFNλ and, interestingly, the ability to degrade IFNλ was associated with intermittent infection status. At closer analysis, the capacity of IFNλ degradation of *P. aeruginosa* isolated from chronically infected patients clustered into two groups. Several reasons can be accounted for this observation. First, staging of patients into chronic or intermittent can be challenging for the clinician and is sometimes not entirely correct. Therefore, scientists search for additional biomarkers of chronicity because treatment of the patients is based on this classification (54). Moreover, CF patients could be colonized by several *P. aeruginosa* strains which do not always display the same phenotype (26, 53). Nevertheless, median IFN degradation activity was significantly decreased in chronic patients, therefore we conclude that chronic patients might have a lower risk of virus infection compared to intermittently infected patients. As discussed before, chronic patients have higher anti-AprA in the serum and this together with decreased protease activity might account for the lower infection rate seen in infected patients.

In order to further investigate a potential link between respiratory viruses and *P. aeruginosa* infections, we screened respiratory material of CF patients using a multiplex PCR based assay. In line with a similar study, we could detect mainly human rhinovirus and to a much lesser extent RSV, Influenza-A/B, Adenovirus or Parainfluenza Virus (55, 56). Interestingly, CF patients intermittent infected with *P. aeruginosa* had a higher risk for hRV infection (Odds ratio = 2.374) and displayed higher virus loads in the sputum compared to *P. aeruginosa* negative or chronically infected patients. However, this observation could just be made if material of the lower respiratory tract (sputum) was analyzed. If also samples from the upper airways (nose, throat) were considered no statistical difference between all groups could be seen. Considering that hRV normally infects the upper respiratory tract, increased detection rates in the lower respiratory airways in *P. aeruginosa* positive individuals might be an indicator for a higher disease burden of hRV infection, similar to the situation in asthmatic or COPD patients (20).

*Pseudomonas aeruginosa* infections have been associated with a worse outcome in CF and with an increased exacerbation rate (10, 57). Interestingly pulmonary exacerbation in CF has been associated with respiratory viruses (13, 14, 20, 55), which might indicate an interplay between respiratory viruses and *P. aeruginosa* infections. This might be more important at early stages of CF when *P. aeruginosa* infections are not yet chronic. In addition, respiratory virus infections have been associated with the conversion of intermittent infections to chronic infections with *P. aeruginosa* (22, 23) caused by increased biofilm growth of *P. aeruginosa* due to increased iron concentrations in the lung (58). Thereby *P. aeruginosa* would directly benefit from a viral infection. Bacterial superinfections of respiratory viral infections are much better described in the literature than viral superinfections and involvement of interferon λ in this scenario has been suggested as well (59). However, several studies show that gut bacteria are able to foster enteric virus infections (60, 61) even though the underlying mechanisms are different (62, 63). In addition, AprA or another LasR dependent protease might directly modify RSV or hRV virions thereby increasing their infectivity, similar to reoviruses (64).

Taken together we could show that *P. aeruginosa* is able to suppress the antiviral response of bronchial epithelial cells by the direct degradation of IFNλ. This degradation was dependent on the quorum sensing transcription factor LasR and the protease AprA. In addition, the infection status of CF patients was associated with the potential to degrade IFNλ and with presence of respiratory viruses in sputum. Therefore, we conclude that interfering with the antiviral response might lead to an increased susceptibility of *P. aeruginosa* infected CF patients for respiratory viruses causing respiratory exacerbations or foster the conversion of intermittent to chronic *P. aeruginosa* infections.

## Materials and Methods

### Materials

RPMI 1640 medium was purchased from Biochrom (Berlin, Germany). FCS was from Life Technologies (Carlsbad, CA, USA). Penicillin and streptomycin were from PAA Laboratories (Pasching, Austria). PBS was obtained from PAN-Biotech (Aidenbach, Germany). EBSS was from Sigma-Aldrich (Saint Louis, MO, USA). Bronchial epithelial basal medium (BEBM) and supplementary growth factors were all from Lonza (Walkersville, MD, USA). PureCol (type I bovine collagen solution, 3 mg/ml) was from Advanced BioMatrix (San Diego, CA, USA). Soybean trypsin inhibitor (STI), DNase I, and Protease XIV were from Sigma-Aldrich. Primary antibodies detecting p-STAT1 and actin were all from Cell Signaling Biotechnology (Frankfurt, Germany). IFNλ antibody was from abcam (ab38569, Cambridge, UK).

### Cell Culture

Human bronchial epithelial BEAS-2B cells were cultured in RPMI growth medium, supplemented with 10% FCS, 1% penicillin/streptomycin at 37°C in a humidified incubator at 5% CO_2_. Human primary bronchial epithelial cell cultures (primary HBE) were bought from Lonza (Visp, Switzerland) or isolated from biopsies from individuals treated by lobectomy because of non-small-cell lung carcinoma at the Thoraxklinik Heidelberg. Ethics approval (S-381/2014) was obtained from the regional Ethics Committee at the University of Heidelberg, and all study participants provided a written informed consent. Two methods of bronchial epithelial cells isolation were used (65, 66): Briefly, the obtained biopsies were washed with cold EBSS and cleaned from any additional connective tissue and mucus. The segments were then cut open and incubated on a shaker at 4°C overnight in 9 ml EBSS including 1 ml digestion solution (DS, 10x: 0.01% DNase I, 1% Protease XIV in sterile PBS). On the next day, 1.1 ml FCS was added to the solution to terminate digestion. The epithelium was scraped into the digestion medium using a sterile scalpel. The biopsies were additionally washed with EBSS. All epithelial cells from scraping were collected by spinning at 4°C at 500 × g for 5 min. Cell pellets were resuspended in 10 ml of warm BEGM (Lonza) and cells were then seeded into collagen-coated (3 mg/ml PureCol, Cellsystems, 1:75 in ddH_2_O) prewarmed 10 cm culture dishes.

Additionally, isolation using an outgrowth method was performed: Bronchial segments were cut into 2–3 mm^3^ pieces and placed into collagen-coated 6-well-plates. These pieces served as a source of primary cells and grown out epithelial cells were transferred into collagen-coated 10 cm dishes as soon as they reached 70% confluence. Primary cell cultures were maintained at 37°C in a humidified incubator at 5% CO_2_. Culture medium (BEBM + Supplements) was changed every 2–3 days. Cells were passaged when reaching 70–90% confluency. After 2 washes with PBS, they were trypsinized with 2 ml of 0.05% trypsin/EDTA and incubated 5–10 min at 37°C. Cells were then rinsed twice with PBS, harvested and pooled into a tube containing soybean trypsin inhibitor (STI, 1 mg/ml) on ice. After spinning at 500 × g for 5 min, the pellets were resuspended in 10 ml BEGM and cell suspension was split into two new collagen-coated 10 cm dishes.

### *P. aeruginosa* Strains and Virus Infections

Various *P. aeruginosa* strains (Table 1) were cultured in lysogeny broth (LB) broth overnight at 37°C, shaking at 200 rpm. The culture was then diluted 1:50 in RPMI-1640 medium and grown for 5 days at 37°C, shaking at 200 rpm, in order to activate quorum-sensing and virulence factor secretion. On day 5, the medium was centrifuged for 10 min at 4,200 rcf and passed through a 0.2 μm filter. This medium was used as conditioned medium (CM). For each experiment at least two different CM were used. A RPMI-1640 control was cultured and filtered alongside the cultured *P. aeruginosa* strains. 100,000 BEAS2B or primary cells were seeded in 24-well-plates in BEAS2B medium. The following day, the cell medium was changed to infection medium, which was RPMI-1640 supplemented with 2% FCS, 1% P/S, 25 mM HEPES, and 0.075% NaHCO_3_. Twenty-four hours later, BEAS2B were pretreated with CM (20% v/v) for 1 h at 37°C, 5% CO_2_. Cell medium was then removed, and respiratory syncytial virus (RSV) or rhinovirus (RV1B) at a multiplicity of infection of 1 (MOI 1) (stocks prepared in house) in combination with fresh CM was added to BEAS2B cells. After 1 h at room temperature, shaking at 30 rpm, medium was removed, cells washed twice with warm 1X PBS, and infection medium added to the cells. Fresh CM was once again added to the cells as indicated above. Cell culture supernatant was collected at the indicated times and cells were lysed in RNA lysis buffer.

**Table 1:** Strain descriptions.

**No**.	**Strain**	**Description and characteristics**	**References**
1	PAO1	*Pseudomonas aeruginosa* wild type	DSMZ 22644
2	Boston	*Pseudomonas aeruginosa* wild type	ATCC 27853
3	PA14	*Pseudomonas aeruginosa* wild type	DSMZ 19882
4	PA7	*Pseudomonas aeruginosa* wild type	DSMZ 24068
5	CF1.1	CF patient isolate	(6, 40)
6	CF1.2	CF patient isolate, clonally related to 5	(6, 40)
7	CF2.1	CF patient isolate	(6, 40)
8	CF2.2	CF patient isolate, clonally related to 7	(6, 40)
9	CF3.1	CF patient isolate	(6, 40)
10	CF3.2	CF patient isolate, clonally related to 9	(6, 40)
11	CF4.1	CF patient isolate	(6, 40)
12	CF4.2	CF patient isolate, clonally related to 11	(6, 40)
13	CF1	CF patient isolate	(6, 40)
14	CF1ΔlasR	CF1 lasR deletion mutant	(6, 40)
15	CF2	CF patient isolate	(6, 40)
16	CF2ΔlasR	CF2 lasR deletion mutant	(6, 40)
17	CF3	CF patient isolate	(6, 40)
18	CF3ΔlasR	CF3 lasR deletion mutant	(6, 40)
19	CF4	CF patient isolate	(6, 40)
20	CF4ΔlasR	CF4 lasR deletion mutant	(6, 40)
21	CF5	CF patient isolate	(6, 40)
22	CF5ΔlasR	CF5 lasR deletion mutant	(6, 40)
23	PA14	*Pseudomonas aeruginosa* wild type parental strain of 24, 25, 26, 27	(43, 67)
24	PA14ΔAprA	PA14 transposon insertion mutant, ID23768	(43, 68)
25	PA14ΔLasA	PA14 transposon insertion mutant, ID35267	(43, 68)
26	PA14ΔLasB	PA14 transposon insertion mutant, ID31938	(43, 68)
27	PA14ΔPrpL	PA14 transposon insertion mutant, ID37740	(43, 68)

### Western Blotting

BEAS2B cells were stimulated as indicated, subsequently washed with PBS, and lysed in Laemmli buffer [400 mM Tris–HCl, pH 6.8, 20% (v/v) β-mercaptoethanol, 40% (v/v) glycerol, 8% (w/v) SDS, and 0.4% (v/v) bromophenol blue]. After incubation for 10 min at 98°C, equal amounts of lysates were fractionated by 10% polyacrylamide gel (SDS-PAGE) and electrotransferred to Nitrocellulose membranes by a semidry blotting procedure [buffer: 25 mM Tris, 192 mM Glycin, 10% (v/v) methanol; 2.5 mA/cm^2^ for 1 h 15 min]. Blocking of unspecific binding was performed using 5% BSA solution in 1× TBST [1× TBS, 0.05% (v/v) Tween-20] for at least 1 h. Membranes were stained with antibodies against pY-STAT1 (Tyr701, #9167), STAT1 (#9172), β-Actin (#4970) (Cell Signaling, Leiden, Netherlands; 1:1,000) overnight at 4°C. After three 10 min washing steps in 1 × TBST at room temperature, blots were incubated with secondary antibodies for 1 h at RT [HRP-linked anti-mouse or anti-rabbit (Cell Signaling, Leiden, Netherlands)], followed by additional three 10 min washing steps in 1× TBST at room temperature. Proteins were detected using an enhanced chemiluminescence system (Western lightning^™^ plus ECL, Perkin-Elmer, Rodgau, Germany). Gels were imaged digitally, and contrast adjustments were applied to all parts of a figure. The prestained molecular weight marker was imaged separately (using transmitted light) and aligned to the digital images of the blots. The ladder is represented on the blots as black bars. Where indicated, membranes were stripped and reprobed. Densitometry was performed using ImageJ software (National Institutes of Health).

### RNA Isolation and Quantitative RT-PCR

Total cellular RNA was isolated using peqGold Total RNA Kit (peqlab Biotechnology, Erlangen, Germany) according to the manufacturer's standard protocol. RNA isolation included DNase digestion using an RNase-free DNase set (Qiagen, Hilden, Germany). In order to perform quantitative RT-PCR, total RNA was first reverse transcribed into single stranded cDNA using High Capacity cDNA RT Kit (Applied Biosystems, Foster City, CA). For RT-PCR analysis, 2 μl of cDNA (diluted 1:4) was used as a template in a final reaction volume of 15 μl, combined with SYBR^®^Green PCR Master Mix Fast (Applied Biosystems) and corresponding primers (Table 2). The analysis was performed on a StepOne Plus RT-PCR platform (Applied Biosystems) in 96-well-format. Each gene was measured in duplicates of each cDNA sample. The baseline and threshold values were detected automatically and the Ct values of the endogenous constitutively expressed reference gene (ACTB) were subtracted from the determined Ct values resulting in a –ΔCt for each target gene, which was then used to calculate the relative expression, rE = 2^−Δ*Ct*^. To control reaction specificity, all measurements included samples without the reverse transcriptase enzyme (noRT). Melting curves were used to prove specific amplification. Fold induction was calculated of the ratio of rE_treated_/rE_Ctr_.

**Table 2:** Primer sequences.

**Name**	**Direction**	**Sequence**
MX1	Fw	CTGCACAGGTTGTTCTCAGC
	Rev	CCAAGGTCCACCGTGATTAAC
OAS1	Fw	TGTCCAAGGTGGTAAAGGGTG
	Rev	CCGGCGATTTAACTGATCCTG
Rv1b	Fw	CTAGCCTGCGTGG
	Rev	AAACACGGACACCCAAAGT
RSV	Fw	GATATGCCTATAACAAAT
	Rev	GATACTGATCCTGCATT
hIFNa1	Fw	CAGAGTCACCCATCTCAGCA
	Rev	CACCACCAGGACCATCAG
hIFNa2	Fw	CTGGCACAAATGGGAAGAAT
	Rev	CTTGAGCCTTCTGGAACTGG
hIFNb	Fw	CGCCGCATTGACCATCTA
	Rev	GACATTAGCCAGGAGGTTCTCA
hIFNL1	Fw	GGACGCCTTGGAAGAGTCACT
	Rev	AGAAGCCTCAGGTCCCAATTC
hIFNL2/3	Fw	CTGCCACATAGCCCAGTTCA
	Rev	AGAAGCGACTCTTCTAAGGCATCTT

### Measurement of Cytokine Secretion

Sandwich enzyme-linked immunosorbent assay (ELISA) was performed using commercially available kits (eBioscience, human IL29 ELISA) to determine the amount of secreted human IFNλ in the cell-free supernatants of stimulated cells. Samples were tested in duplicates and the assays were performed according to the manufacturers' instructions. Cytokines were detected by measuring the absorbance at 490 nm with a 650-nm reference in a photometer (Sunrise reader, Tecan, Salzburg, Austria). Cytokine concentrations were calculated according to a standard dilution of the respective recombinant cytokines using Magellan V 5.0 software (Tecan, Salzburg, Austria).

### Protease Activity Assay, Zymography

CM was treated with non-reducing Western blot sample buffer and separated by SDS-PAGE (10% polyacrylamide gel containing 0.1% gelatin). The gels were washed twice with 2.5% Triton-X-100 for 15 min followed by an overnight incubation at 37°C in substrate buffer (10 mM Tris, pH 8.0, 10 mM CaCl_2_, 1 μM ZnCl, 150 mM NaCl). Subsequently the gel was stained with 0.5% Coomassie blue in acetic acid:isopropanol:dH_2_O (10:30:60) and pictures were taken after destaining with dH_2_O. In order to determine total protease activity in CM a sterile 6 mm filter disk was added on skim milk agar (1.5%) and 10 μl of CM was added. Following incubation at 37°C the diameter of clearance (as an indication of protease activity) was measured.

### *In vitro* Degradation of IFNλ

IFNλ was incubated with CM for the indicated time at 37°C and 5% CO_2_ in the dark. As a loading control IFNλ was added to RPMI only. Reactions were stopped by adding western blot sample buffer and heat treatment (95°C, 10 min). Subsequently samples were used for western blot analysis. Quantification was performed using ImageJ and samples were normalized to the input control.

### Patients and Virus Detection

Studies including samples from CF patients were approved by the Ethics Committee of the University of Heidelberg (study number S-370/2011). Informed written consent was obtained from the patients, parents, or legal guardians of all subjects. Airway samples from CF patients (sputum, throat swab) were obtained during routine visits at the CF Center at the University Hospital Heidelberg as previously described (54). The diagnosis of CF was based on established diagnostic criteria. Samples were used for isolation of *P. aeruginosa* strains (*n* = 51) and sputum, nose or throat swabs were analyzed for virus infection by multiplex PCR (Seegene, Korea, Allplex^TM^ respiratory panels 1–4) or by targeted RT-PCR after RNA extraction using TRIZOL and cDNA synthesis as described above. Infection status with *P. aeruginosa* (intermittent or chronic) was classified according to the following definition (69): Intermittent infection was defined as positive microbial culture of *P. aeruginosa* in at least one and <50% of the samples in the last 12 months and no detection of anti-*Pseudomonas* antibodies (against alkaline protease, elastase, and exotoxin A). Chronic infection was defined as persistent culture presence of *P. aeruginosa* for at least 6 months, or less when combined with a positive finding (titer >1,250) of two or more antibodies.

### Statistical Analysis

All experiments were repeated three times unless stated otherwise. Data are shown as mean + SD. Statistical significance of comparison between three or more unmatched groups was determined by one-way ANOVA and if multiple comparisons were performed, two-way ANOVA was used (both including Bonferroni post-test). Statistics on quantitative PCR (qPCR) data were performed on previously log-transformed data to achieve a normal distribution. All statistical analyses were done using GraphPad Prism (GraphPad 5.00 and 6.05, San Diego, USA) software. Significant differences were considered at ^*^p < 0.05, ^**^p < 0.01, and ^***^p < 0.001 as compared to the control condition. n.s., not significant.

## Data Availability Statement

All datasets generated for this study are included in the article/Supplementary Material.

## Ethics Statement

The studies involving human participants were reviewed and approved by the Ethics Committee of the University of Heidelberg. The patients/participants provided their written informed consent to participate in this study.

## Author Contributions

MS and AD conceived and designed the experiments. MS, JK, and LB performed the experiments. SB and MS analyzed the data. FL, HZ, and DN provided study material. MS, AD, MM, and DN conceptualized the study. MS and AD wrote the paper.

### Conflict of Interest

The authors declare that the research was conducted in the absence of any commercial or financial relationships that could be construed as a potential conflict of interest.
