# Corrigendum: *Pseudomonas aeruginosa* Modulates the Antiviral Response of Bronchial Epithelial Cells

**DOI:** 10.3389/fimmu.2020.01453

**Published:** 2020-07-10

**Authors:** Michael Sörensen, Julia Kantorek, Lauren Byrnes, Sébastien Boutin, Marcus A. Mall, Felix Lasitschka, Heike Zabeck, Dao Nguyen, Alexander H. Dalpke

**Affiliations:** ^1^Department of Infectious Diseases, Medical Microbiology and Hygiene, University Hospital Heidelberg, Heidelberg, Germany; ^2^Laboratory Enders and Partners, Stuttgart, Germany; ^3^Translational Lung Research Center Heidelberg (TLRC), German Center for Lung Research (DZL), University Hospital Heidelberg, Heidelberg, Germany; ^4^Department of Pediatric Pulmonology, Immunology and Intensive Care Medicine, Charité Universitätsmedizin Berlin, Berlin, Germany; ^5^Berlin Institute of Health (BIH), Berlin, Germany; ^6^Institute of Pathology, University Hospital Heidelberg, Heidelberg, Germany; ^7^TI Biobanking, German Centre for Infection Research (DZIF), Heidelberg, Germany; ^8^Thoraxklinik, University Hospital Heidelberg, Heidelberg, Germany; ^9^Meakins-Christie Laboratories, Research Institute of the McGill University Health Centre, Montreal, QC, Canada; ^10^Department of Medicine, McGill University, Montreal, QC, Canada; ^11^Institute of Medical Microbiology and Hygiene, Medical Faculty, Technische Universität Dresden, Dresden, Germany

**Keywords:** *Pseudomonas aeruginosa*, cystic fibrosis, virus, antiviral response, interferon, protease, LasR

In the original article, there was a mistake in Table 1 as published. Three ID numbers of the PA14 transposon mutants were mixed-up (no. 24-26). The corrected Table 1 appears below.

**Table 1:** Strain descriptions.

**No**.	**Strain**	**Description and characteristics**	**References**
1	PAO1	*Pseudomonas aeruginosa* wild type	DSMZ 22644
2	Boston	*Pseudomonas aeruginosa* wild type	ATCC 27853
3	PA14	*Pseudomonas aeruginosa* wild type	DSMZ 19882
4	PA7	*Pseudomonas aeruginosa* wild type	DSMZ 24068
5	CF1.1	CF patient isolate	(6, 40)
6	CF1.2	CF patient isolate, clonally related to 5	(6, 40)
7	CF2.1	CF patient isolate	(6, 40)
8	CF2.2	CF patient isolate, clonally related to 7	(6, 40)
9	CF3.1	CF patient isolate	(6, 40)
10	CF3.2	CF patient isolate, clonally related to 9	(6, 40)
11	CF4.1	CF patient isolate	(6, 40)
12	CF4.2	CF patient isolate, clonally related to 11	(6, 40)
13	CF1	CF patient isolate	(6, 40)
14	CF1ΔlasR	CF1 lasR deletion mutant	(6, 40)
15	CF2	CF patient isolate	(6, 40)
16	CF2ΔlasR	CF2 lasR deletion mutant	(6, 40)
17	CF3	CF patient isolate	(6, 40)
18	CF3ΔlasR	CF3 lasR deletion mutant	(6, 40)
19	CF4	CF patient isolate	(6, 40)
20	CF4ΔlasR	CF4 lasR deletion mutant	(6, 40)
21	CF5	CF patient isolate	(6, 40)
22	CF5ΔlasR	CF5 lasR deletion mutant	(6, 40)
23	PA14	*Pseudomonas aeruginosa* wild type parental strain of 24, 25, 26, 27	(43, 67)
24	PA14ΔAprA	PA14 transposon insertion mutant, ID23768	(43, 68)
25	PA14ΔLasA	PA14 transposon insertion mutant, ID35267	(43, 68)
26	PA14ΔLasB	PA14 transposon insertion mutant, ID31938	(43, 68)
27	PA14ΔPrpL	PA14 transposon insertion mutant, ID37740	(43, 68)

The authors apologize for this error and state that this does not change the scientific conclusions of the article in any way. The original article has been updated.

