# Supplementary material for: Pseudomonas aeruginosa Modulates the Antiviral Response of Bronchial Epithelial Cells
Source: Front Immunol. 2020 Feb 4;11:96. doi: 10.3389/fimmu.2020.00096 (PMC7025480; doi:10.3389/fimmu.2020.00096)
Supplement: Supplementary file 1 [file Data_Sheet_1.PDF]

Supplementary Material

Fig. S1

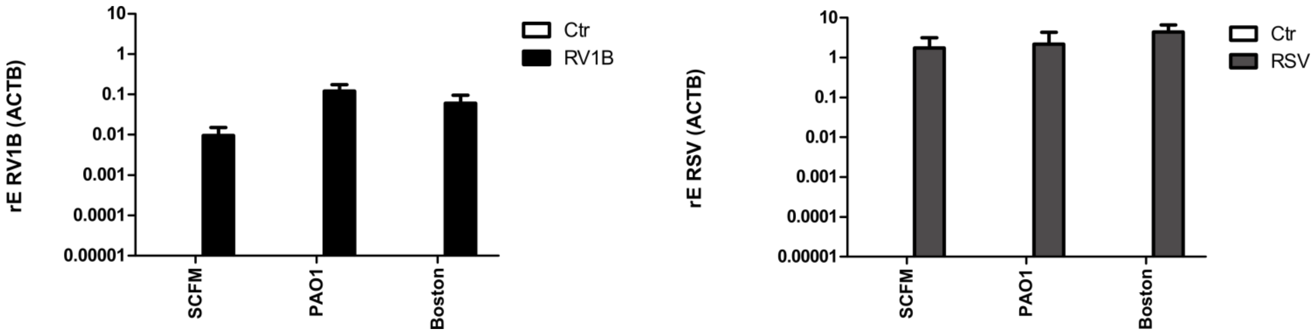

P. aeruginosa has no effect on viral levels. BEAS2B cells were pretreated with conditioned medium of *P. aeruginosa* (PAO1 and Boston) or control medium and subsequently infected with RSV or RV1b. Level of RV1B and RSV RNA were analyzed by qRT-PCR after 14 h.

Fig. S2

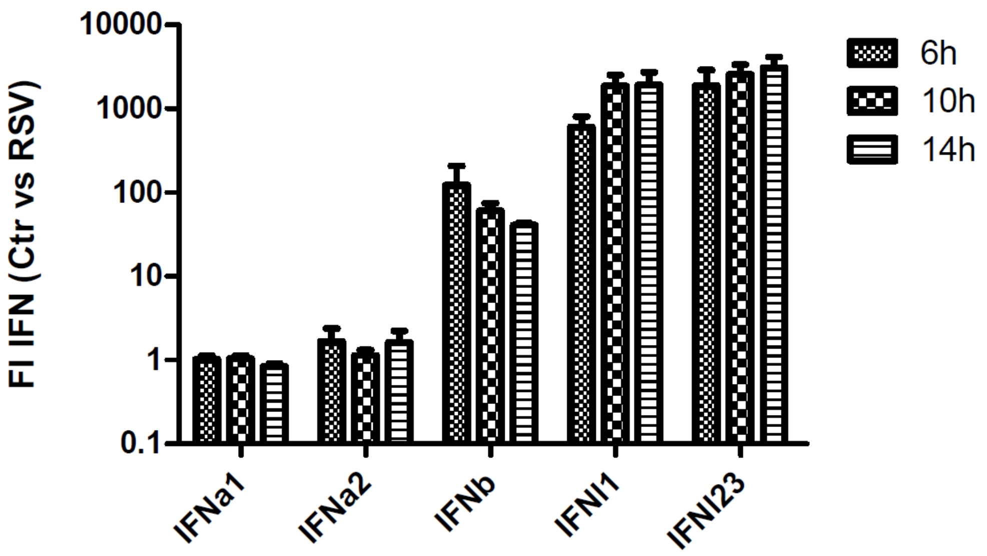

BEAS2B cells were infected with RSV and induction of IFNα (IFNa1/2), IFNβ (IFNb) and IFN λ (IFN1/2/3) mRNA were analyzed by qRT-PCR after indicated time.

Beas2B express type III IFN to a much higher level than type I IFN as reported in many epithelial cells. We did not observe any induction of IFNα in BEAS2B cells and the induction of IFNβ was modest compared to type III IFN and not persistent. In line with this, we could not detect IFNβ protein by ELISA.

Fig. S3

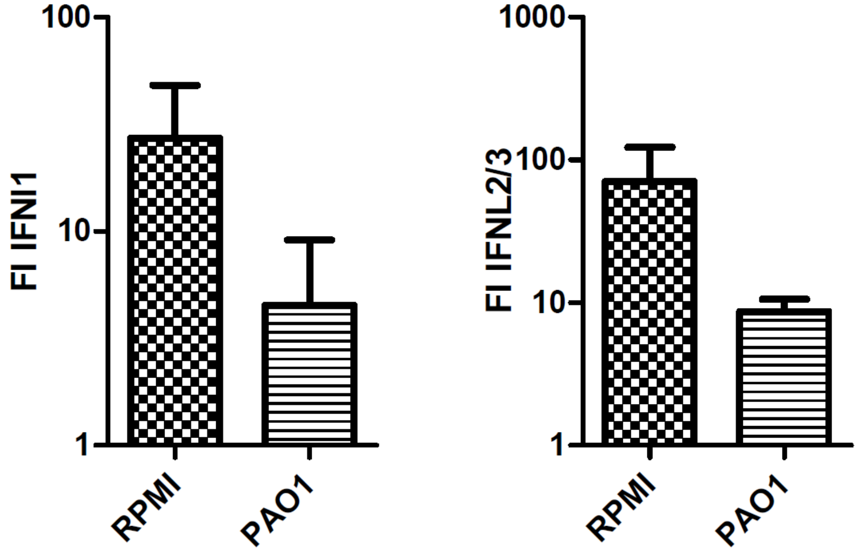

BEAS2B cells were pretreated with conditioned medium of *P. aeruginosa* PAO1 or control (RPMI) medium and subsequently infected with RV1b. Induction of IFNL1 and IFNL2/3 mRNA were analyzed by qRT-PCR after 14 h.

Fig. S4

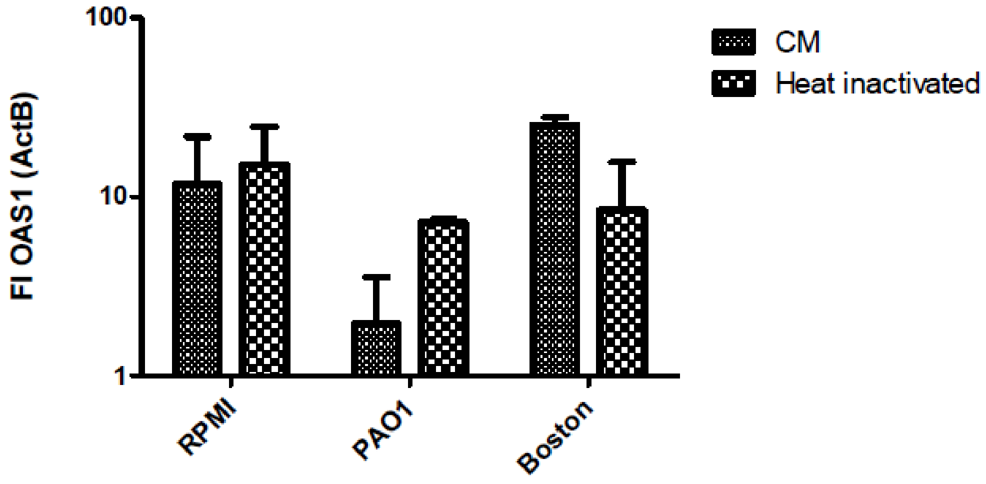

Conditioned media was heated for 10 min at 95°C. Subsequently BEAS2B cells were pretreated with heat treated or non-treated conditioned medium of *P. aeruginosa* (PAO1 and Boston) or with control medium and subsequently infected with hRV. Induction of OAS1 mRNA was analyzed by qRT-PCR after 14 h post infection.

Fig. S5

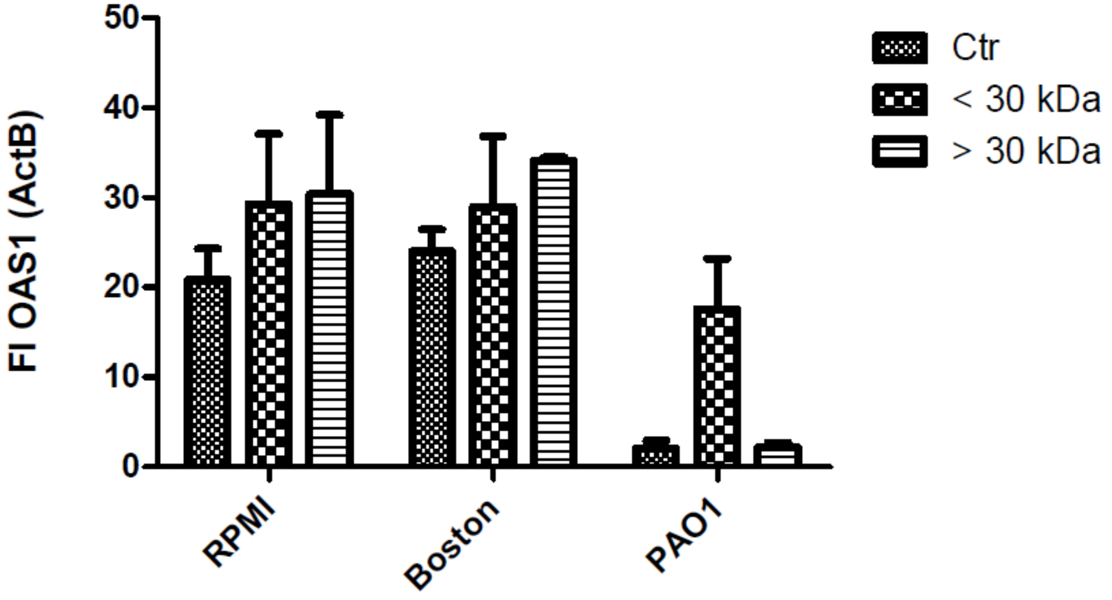

Conditioned media was filtered using a cutoff of 30 kDa. Subsequently BEAS2B cells were pretreated with the fraction larger than 30 kDa, smaller than 30 kDa, non-filtered conditioned medium of *P. aeruginosa* (PAO1 and Boston) or control medium and subsequently infected with hRV. Induction of OAS1 mRNA was analyzed by qRT-PCR after 14 h post infection.

Fig. S6

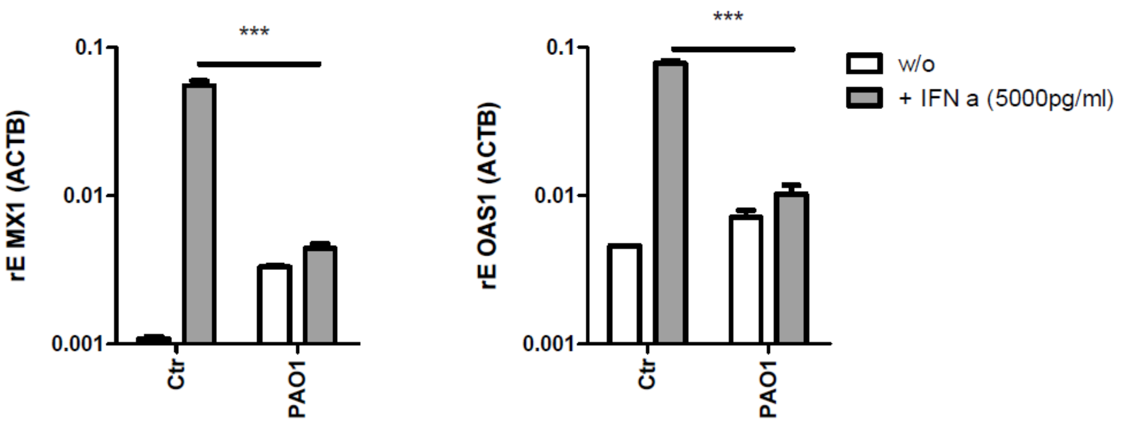

BEAS2B cells were pretreated with conditioned medium of *P. aeruginosa* PAO1 or control medium. After treatment with recombinant IFNα (5000 pg/ml) induction of MX1 and OAS1 were analyzed after 14 h by qRT-PCR.

Fig. S7

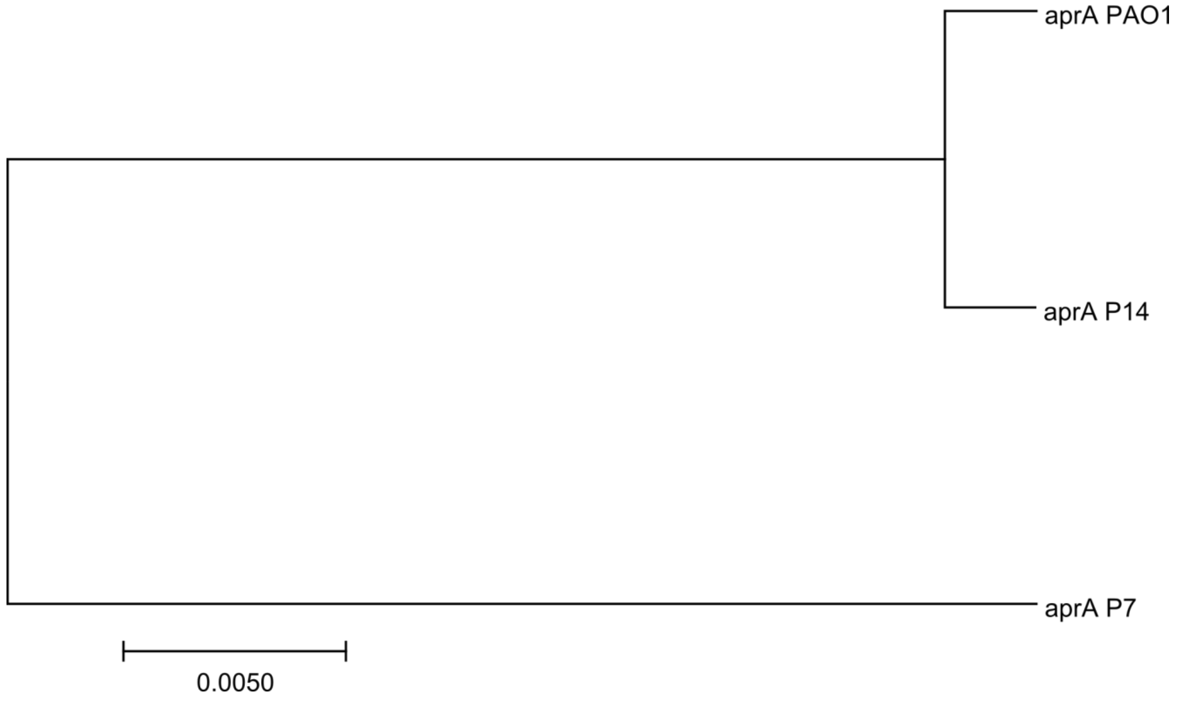

Phylogenetic analyses of AprA of PAO1, PA7 and PA14.
